# Motivations and barriers to engagement with a technology-enabled community wide physical activity intervention

**DOI:** 10.1371/journal.pone.0232317

**Published:** 2020-06-26

**Authors:** Marc Ashley Harris, Diane Crone

**Affiliations:** Cardiff Metropolitan University, School of Sport and Health Sciences, Cardiff, Wales, United Kingdom; University of Edinburgh, UNITED KINGDOM

## Abstract

Previous physical activity interventions have failed to create population change and an alternative approach is needed to support a World Health Organization target of a 15% reduction in global levels of inactivity by 2030. There is growing evidence that gamification-based interventions can reach substantial portions of the community. However, to date, these studies have been predominantly quantitative and as such there is a paucity of research in the area on motivations and barriers to engagement with these programs. Four focus groups conducted with N = 26 players who participated in a gamification-based intervention ‘Beat the Street’ revealed several varied motives to engagement, including collective reward; social influence; game reinvention; exploration; accessibility and awareness. However, several barriers specific to the Beat the Street intervention and outdoor gamification interventions more generally were also identified. This study provides novel insight into the motives which engage individuals into physical activity interventions and the design principles which need to be considered when implementing interventions of this nature.

## Introduction

Despite significant investment in physical activity interventions over the past 20 years, a recent study concluded that 27.5% of adults were insufficiently active, globally, and that levels of inactivity in high-income western countries had increased by over 5% between 2001 and 2016. [1,2] Physical inactivity is now attributable to an estimated 5.3 million deaths globally each year. [3] In England, population level data indicates that 24.8% of adults and 29.0% of children are insufficiently active. [4,5] Further, there are significant and deep-rooted differences in activity levels among different population cohorts. Specifically, women, lower socio-economic groups and people from Black and Asian backgrounds are more likely to live inactive lifestyles. [4] In view of this international concern, the World Health Organization published a Global Action Plan on Physical Activity with an agreed mission to reduce adult and adolescent inactivity levels by 15% from a 2016 baseline, by 2030. [6]

Previous attempts to reduce physical inactivity have focussed on individualistic approaches, such as exercise referral/on prescription programs, clinical populations and community groups and whilst some have shown improvement for specific population groups, they have made little impact at community-wide level. [7–11] In 2017, Schiphorst et al., identified the seven best investments to increase population physical activity levels. These included, 1) Communication and public education, 2) Transport and the environment, 3) Urban design and infrastructure, 4) Healthcare and health education, 5) Education, 6) Community-wide programs, and 7) Sport and recreation. [12] One of these recognised best investments, community-wide approaches, offer the potential to be part of a wider solution to curtailing current projections in physical inactivity by creating local environments that facilitate physical activity across substantial portions of the community. Such approaches aim to create systemic change across multiple sectors and reflect the social-ecological model of health. This model recognises the complex interaction between individual, relationship, community and societal factors and implies action is necessary at multiple levels to achieve behaviour change. Community-wide approaches, such as mass-communication or free community physical activity classes in public spaces, focus on policy, the environment, mass media and supporting individualistic interventions. [13,14]

Interventions which incorporate game elements and technology have been successful at engaging substantial portions of the population. These approaches apply game principles (such as leader boards, feedback, social comparison and co-operation) to encourage people to walk, cycle and generally be more active. Pokémon Go is one example which demonstrates the mass-appeal of these technology supported, gamified approaches. At its peak Pokémon Go had an estimated 45million daily participants worldwide. Whilst this game was never designed to be a public health, or physical activity intervention, it did result in encouraging users to walk, run and explore their environment, as a biproduct of gameplay. Due to both the novelty of mass scale gamification-based approaches and their very recent application, the evidence-base is, to date, sparse. According to Corepal et al. they conclude that gamification interventions have rarely been developed using theoretical frameworks and furthermore, little is known about the perspectives of those that take part. [15]

Whilst the Corepal et al., paper was published in 2018, research in this area is still, to date, sparse. Recent qualitative efforts have attempted to unravel the motivational constructs underpinning the mass-appeal of these approaches. In one study, Shameli et al. found competition incentivised users of a smart-phone walking challenge to increase their activity, however group dynamics (such as pre-competition activity levels, probability of winning, gender balance and inclusion of experienced competition participants) had both positive and negative effects on engagement. [16] Elsewhere, Looyestyn and colleagues found leader boards where more effective at increasing engagement than badges, points and rewards. [17] Research examining why individuals chose to take part, and continue to take part, in online games has found that socialisation, escapism, competitiveness, fantasy and friendship building may be important drivers to participation. [18,19]

### The current study

At a general level, there is a paucity of research investigating engagement with these novel approaches that specifically tackle levels of inactivity. Further, there is an even greater lack of evidence regarding the study of the lived experience of participants from a qualitative perspective. As a response to the gap in the current literature, this study aimed to identify why participants engaged with the intervention, and the barriers to participation they encountered during a community-wide gamification-based physical activity intervention, ‘Beat the Street’.

## Materials and methods

An exploratory qualitative research design was used to explore the motivations underpinning engagement with the intervention. Focus groups and thematic analysis were used to collect and analysis the data for the study.

### Intervention

Beat the Street is a community-wide, gamification-based physical activity intervention. The intervention aims to change levels of physical activity by turning a local area into a game where participants register their walking and cycling journeys by tapping a Radio-frequency identification (RFID) card on RFID readers called ‘Beat Boxes’. Beat Boxes are placed on lampposts throughout the locality, in greenspaces and in places of local interest (such as museums, leisure centres and parks) at half-mile intervals. Points are accrued by distance travelled whereby each time two boxes are touched in under 1 hour a player receives 10-points for themselves and their team. At the end of the game period, highest scoring schools, community groups, and individuals are rewarded with prizes, such as vouchers for sports equipment, craft or book vouchers, or cash prizes. An online portal is used to engage with each participant. This platform allows players to register to take part, select a team they wish to be a part of, provide data on their health and wellbeing, monitor their progress, compare themselves with others and receive up-to-date communications about the game. It has previously been assumed that children and adults compete to see which schools, community groups and individuals can achieve the greatest number of points throughout the 7-week game period, however little is known about the precise motivating components, nor indeed barriers to particpation. [20,21] The initiative is managed centrally by a health-technology company ‘Intelligent Health’ in Reading, UK.

### Procedure

Beat the Street ran between March 15 and May 13 2017 in Wolverhampton, England. Adult participants who took part in the intervention were encouraged to register their RFID card via an online portal which allowed them to select a team to join, such as their child’s school, their workplace or a local charity. During registration participants were asked to complete a range of sociodemographic questions and a validated physical activity questionnaire. [22] Immediately following the intervention registered participants who agreed to be contacted (N = 3315) were sent an email inviting them to take part in a follow-up survey and a £50 prize draw was offered to incentivise survey completion. A total of N = 607 people completed the follow-up survey and a second follow-up email was sent to participants who were either inactive (undertaking 0–30 minutes of activity per week) or low active (undertaking 31–149 minutes of activity per week) before the intervention inviting them to take part in a focus group. Activity levels were calculated using a validated single-item measure of physical activity. [22] All participants were offered £50 cash to take part in the study.

Four focus groups were undertaken with participants (FG 1 n = 7 males, FG 2 n = 6 females, FG 3 n = 6 males and FG 4 n = 7 females) and were organised by gender to ensure participants felt comfortable when discussing their motivations and barriers to activity. Each focus group lasted for approximately 2 hours with participants spending 1.5 hours in their discrete groups before coming together into two larger, mixed gender, groups for a 30-minute reflection and sharing session. The data collection period was completed within 3 weeks of the intervention concluding. Informed consent to be contacted following the game period was obtained electronically via the online portal and written consent was obtained prior to each focus group. Ethical approval was granted by Cardiff Metropolitan University, Psychology Research Ethics Committee (Ref 8405). Participation in the intervention was entirely voluntary and participants could choose to partake in the intervention without being contacted in future, providing data at baseline and/or follow-up and taking part in a focus group post-intervention.

Each focus group was guided by an identical discussion guide. The discussion guide was developed following extensive consultation between the researchers, intervention deliverers (Intelligent Health) and a review of past literature exploring the motivations to engagement with gamification-based interventions. The focus groups were split into 5 sections. First, participants were asked to introduce themselves and discuss their hobbies, what they like to do to relax and the words they associate with sport, exercise and activity. Second, participants were asked their general drivers and barriers to physical activity and specifically what makes a good experience and what makes a bad experience. Third, participants were asked to discuss their direct experience of Beat the Street, their reasons for taking part, the initial prompt, how they found out about it, what made them sign-up and if they got what they hoped to achieve out of the game. Fourth, the groups were asked to discuss any impact Beat the Street had on their attitudes and behaviours, if they felt it had led to change and if any change will continue. Finally, once the four groups were combined into 2 groups with mixed genders, they were asked to share what they had discussed previously with each other and to visualise and discuss what a future intervention would look like in an ideal word.

### Sample and participants

Beat the Street was delivered as a pilot intervention on behalf of Sport England, a national non-departmental public body, and City of Wolverhampton Council. Wolverhampton was chosen as an important location for this pilot due the area possessing high levels of inactivity and multiple deprivation. The intervention engaged a total N = 25,790 people. None of those participants, nor in fact in any of the previous intervention participants to date, had been invited to participate in a qualitative investigation into understanding motivations for engagement. Therefore, a selection were invited to take part, of which N = 26 players were recruited to take part in the study. Participants that took part in the focus groups had a mean age of 44, which ranged from 25 to 60, and there was an even distribution of males and females (50:50). Of all adults who took part in the intervention (n = 10,574) 80% were aged between 19 and 49 and 71% were female. As such, the focus groups had strong representation of intervention participants more broadly.

### Method of analysis

The focus groups were audio recorded and transcribed following the completion of the data collection period. Data were analysed using thematic analysis and followed the six stage principles outlined by Braun and Clarke. [23,24] In step 1, familiarization with the data was achieved by the lead researcher collecting, transcribing, reading and re-reading transcripts and creating notes of initial themes of interest throughout the process. Step 2, generating initial codes, was achieved by systematically coding, line-by-line, interesting features within the data and each transcript was read twice during the coding process. During step 3, codes were collated into themes and supporting quotes were attached to each emergent theme. Step 4 involved a review of each theme, a refinement of themes and a retirement of previous themes without adequate data to support their claims. During this process the lead research listened to the audio recordings again to confirm if each theme accurately reflected the context in which they were discussed. At stage 5, each theme was further refined, provided a definition, grouped into superordinate themes and given a name of reference within this paper. The thematic analysis culminated in stage 6 where each theme and supporting evidence (in the form of quotes) was shared among the research team. At this stage, leading quotes were selected which best elucidated the topic of interest, provided the lived experiences of the participants to be evident in the findings, and were then used to explain each theme. Reflexivity was assured through the interplay of the systematic, iterative approach to data analysis and discussion between the two researchers in the latter stages of the analysis process and theme confirmation.

## Results and discussion

Through the process of thematic analysis, [23] seven themes were identified in the data relating to engagement and barriers to participation. These included: (1) collective reward; (2) social influence; (3) game reinvention; (4) exploration; (5) accessibility; (6) awareness; and (7) barriers to engagement. These themes (superordinate) and their subthemes (subordinate) are presented in Table 1 with participant quotations and are elaborated upon below with supporting explanation and further quotations. Pseudonyms have been used to protect the identity of participants.

**Table 1:** Motivations and barriers to engagement with Beat the Street.

Superordinate Theme	Subordinate Themes	*Evidence*
**1. Collective Reward**	**Points**	*“I went to three markets I hadn’t been to in years*. *They had double points boxes*.*”–Female*, *involved directly*
	**Charity**	*“It was about Wolverhampton benefiting from the total*. *Compton Hospice would get a donation*. *Work did say about friendly competition but for me it was more personal*, *health and Compton*.*” Female*, *involved directly*
**2. Social Influence**	**Child reinforcement**	*“Doing the Beat the Street*, *me and my daughter went from one to another*. *She's six*. *For her and me to walk a distance*, *it was good*. *She loved it*. *She said it was really good*. *For her to do it*, *we had a good feeling about it*.*”–Male*, *involved through child(ren)*
		*“You’d never catch my dad walking but he was doing it because of my sister and niece*.*”-Female*, *involved through child(ren)*
	**Community reinforcement**	*“I became addicted to it and wanted to tick off as many as I could*. *I liked going to new places and meeting people*. *It was so friendly*. *Watching other families take part in it*, *it was such a good feeling*.*” -Female*, *involved directly*
		*“More or less the same*. *Every night we’d have people ringing us*, *‘Are you going out tonight*?*’ My dog loved it*. *Walking the dog has never been my job*, *but that’s something that’s continued*.*”–Female*, *involved directly*
	**Family Reinforcement**	*“Family was a big one for me*. *Engaging in any task on your own*, *you can do it but you feel separated*. *When we did it together it was more motivating*. *It kept us all going*. *We all have to stay connected to get something done*.*”–Male*, *involved through child(ren)*
	**Parental reinforcement**	*“She [daughter] dragged me to Beat the Street*, *I drag her to spin*. *We couldn’t walk for the first week*, *we were in agony but it’s got easier*.*”–Female*, *involved through child(ren)*
**3. Game Reinvention**	**Role playing**	*“To go with them*. *Our kids*, *we don't want them to get lost*. *So it’s good for me to show them the local areas and show them how to map read*. *We changed it into an army thing*. *We made it fun*.*”–Male*, *involved through child(ren)*
	**Escapism**	*“For the kids it’s like a mini adventure or a treasure hunt*. *My daughter found adventure in the whole thing*. *I got excited about it as well*. *It wasn’t dull*. *For such a simple thing*, *it became an adventure*. *It was really good*.*”—Female*, *involved through child(ren)*
	**Challenge**	*“Just to get out*. *It was twenty minutes then half an hour then 40 minutes*. *I managed to do five hours non-stop on the art gallery day*.*”–Female*, *involved directly*
		*“I’ve set myself more goals*. *I’m going to walk in Hawkstone Park near Shrewsbury*. *I want to do Snowdonia someday*. *I’m trying to aim high*.*”–Female*, *involved directly*
**4. Exploration**	**Orienteering**	*“I did round Newbridge Way*. *When I visited my mum there I looked at the map and found some boxes so did a route there*.*”–Female*, *involved directly*
		*“My little boy was looking at the map*, *marking it off and circling where he’d been*.*”–Female*, *involved through child(ren)*
		*“Being involved in a successful day*, *it becomes habit*. *You come home thinking you’ve had a good day*. *So that's an addictive feeling*. *Once we knew we could map up which ones we were going to*, *then we could do that on the way to other places*.*” -–Male*, *involved through child(ren)*
	**Collection**	*“Once I got the map and saw where they were*, *my objective was to visit every one*. *I did that in about three weeks then had a week off*. *Then I picked up again to accumulate points”–Male*, *involved directly*
		*“There's the collecting side of it*. *With Beat the Street*, *you're collecting posts*. *It’s a tally*. *You can show how much you’ve achieved”*.*–Male*, *involved through child(ren)*
		*“It was trying to get around as many as we could*.*”—Male*, *involved directly*
		*“We didn’t want it to beat us*. *We wanted to beat it*. *We tried to get as many as we could*.*”–Male*, *involved through child(ren)*
	**Discovery**	*“Getting to discover places I didn’t know*. *We did Cupcake Lane and the abandoned station is a teashop*. *One day I thought*, *‘Let’s try going a little further*.*’ I hadn’t realised there was a massive park at the back of Newbridge*. *I walked all around there and found the Wolves training ground*.*“–Female*, *involved directly*
		*“I went to parts of Wolverhampton I’d never been to before*. *Why would I go for a walk the other side of town and discover canals and cemeteries*?*”–Female*, *involved directly*
**5. Accessibility**	**Low physical demand**	*“It gave you the time with your family*, *doing something different*. *You weren’t likely to get an injury*.*”–Male*, *involved directly*
	**Convenience**	*“There's nothing more accessible than walking around*. *Clubs usually have a membership or some kind of cost involved*. *This is accessible for anyone*.*”–Male*, *involved directly*
		*“You’ve got family and work commitments*. *You can play the game on your doorstep*.*” Male*, *involved directly*
	**Active Travel**	*“I travel to town from where I live to do my shopping and I usually catch the bus*. *Since then I've been walking rather than catching the bus*. *I walked a route to the town centre that went past all the boxes*. *It’s a bit out of the way but doesn’t take that much longer*, *and I still walk that way*.*” -Male*, *directly*
		*“If I go to Asda in Wolverhampton I do walk down there and catch the bus back*. *With Beat the Street*, *there was the incentive to carry my shopping and walk back*.*”–Female*, *involved through child(ren)*
**6. Awareness**	**Exercise**	*I’ve started swimming as well as continuing with the walking*. *I want to get back into Zumba*.*”- Female*, *involved directly*
		*“We’ve started gardening again and have made a little veggie patch*.*”—Female*, *involved directly*
		*“I went back to the gym*. *It prompted me to think about how important it was*. *We live too far from the school to walk the whole way*. *With Beat the Street we started walking partway*. *I felt healthier*, *more toned and it reminded me how I used to feel*. *I never used to care about people looking at me and I’d started to care so I wanted to get back to how I used to feel*.*”–Female*, *involved through child(ren)*
		*“I hadn’t ridden a bike for years*. *It prompted me to do it*. *My mum lives probably just less than a mile away*. *Instead of driving I get on my bike*.*”–Female*, *involved through child(ren)*
	**Nutrition**	*“I’ve recently changed my attitude to the way I’m eating*. *I used to think*, *‘What’s the point in doing any exercise if I’m not eating right*?*’ Now I think I need to eat right because I’ve been exercising*. *If you eat fish and chips after exercising you realise you’re undoing the good work*.*”—Female*, *involved directly*
		*“My attitude towards food has changed*. *I eat better*. *I’d rather go in and have a bowl of fruit salad*. *I do miss Cadbury’s though*!*”–Female*, *involved directly*

**7. Barriers to Engagement**	**Technology**	*“Sometimes the little ones would fight to tap my card*. *Sometimes they didn’t all work*. *Some of the boxes didn’t work*.*” Female*, *involved directly*
	**Safety**	*“If I was on my own I felt a bit uncomfortable about going to certain places*. *Maybe have groups going to some of the boxes*.*”–Female*, *involved directly*
		*“I think they need to think about where the boxes are situated*. *The canal is lovely for adults but not so good for children*.*”–Female*, *involved directly*
	**Visibility**	*“The boxes were the same colour as the lamppost and you'd go past it*. *Put fluorescent tape on it*.*”–Male*, *involved directly*
		*“I had that*. *One of them was in the trees*. *I've seen people come down the road looking for the box and not paying attention to the road*.*”–Male*, *involved directly*
	**Practicality**	*“I don’t know how the boxes were put together*. *There seemed to be a lot in one place and not many in others*. *I don’t know what the rationale was*. *More of an even spread*. *Maybe look at who’s used it over the last two years*. *The maps were complicated*.*”–Female*, *involved directly*
		*“Some of them were too high up*, *and the kids want to swipe them themselves*.*”–Male*, *involved through child(ren)*
	**Understanding**	*“Getting the points*. *I used to feel sorry for them in the morning outside the school*. *They’d tap their machine before they went into school*. *They weren’t getting the points*. *You have to tap a machine and then tap another one*. *They thought if they tapped the machine and then went into school they’d get points*. *My motivation was to get points for those children who couldn’t get them*.*”–Female*, *involved through child(ren)*

### Collective reward

Unsurprisingly, given the nature and design of the intervention, extrinsic motivations were a key incentive underpinning engagement with the program. These rewards, however, were predominantly social and altruistic in nature with participants wanting to ‘win’ for others, such as a community group or school.

For instance, Andrew: “My wife wanted to win the money for her walking group. We got everyone to sign up for it” and Rebecca: “I used to feel sorry for them (children) in the morning outside school, they’d tap their machine before they went into school. They weren’t getting the points. You have to tap a machine and then tap another one. My motivation was getting points for those children who couldn’t get them”. Stacey shared a personal story about how the collective charity prize was a key driver to her participation, explaining “It was about Wolverhampton benefiting from the total. Compton Hospice would get a donation. Work did say about friendly competition but for me it was more personal, health and Compton.”

### Social influence

The social nature of Beat the Street was the overarching catalyst for participation. Whether family, friends, colleagues, or the Compton Hospice charity, participants wanted to take part in an activity with other people, and for other people.

Gareth and Alan: “Family was a big one for me. Engaging in any task on your own, you can do it, but you feel separated. When we did it together it was more motivating. It kept us all going. We all have to stay connected to get something done.” and “It’s for me and my son. I like boxing but I have arthritis. This Beat the Street made me get up and now I do all sorts with my son. I enjoy it. I’m a van driver so I’m sat down all day, but this got me doing something different. Now I do more things.” respectively. However, the social coercion (often referred to as ‘pester power’) [22,23,25] by parents and children operated in a reciprocal nature. For Claire the emphasis was on setting an example to her son, explaining “I was really enthusiastic. I became evangelical. I think it was great. My motivation was to encourage my son to be more active and walk more.” In contrast to Lucy (also involved through her child(ren), where it was the children inspiring the wider family, “The kids motivated you to have that family time. It was downtime for me from work. When you see your kid laughing and smiling and you haven’t forced yourself to do it, it’s just total relaxation.”

### Game reinvention

The scheme had a very simple underlying concept where participation merely involved tapping a card on a physical box. This simplicity allowed individuals to invent their own concept of participation. For some, particularly children, they could incorporate a fantasy, role-play element to the ‘collection’ of boxes. Whereas others used the program to track their activity, set targets and compete with friends.

Rhys: “I used Beat the Street with the guys I work with in therapy as a benefit for mental health. They’re adults and they love it. They want to carry on doing it even though it’s finished. It really helps them.” and Suzanne: “Rather than eating lunch in the office, a couple of friends and I made an effort to go out and walk. It meant we could go out and have a chat. We all have Fitbits so we were competing.” This was in contrast to Maggie who expressed how the children where influential in shaping the game with their imaginations, “For the kids it’s like a mini adventure or a treasure hunt. My daughter found adventure in the whole thing. I got excited about it was well. It wasn’t dull. For such a simple thing, it became an adventure. It was really good.”

### Exploration

The participants articulated a strong sense of satisfaction from exploring their local area, whether that be areas of blue or greenspace, local markets or neighbouring streets. Participants were shocked to discover new features of the environment they thought they were previously familiar with. The game appeared to give them the license, or purpose, to explore the areas where they lived and worked, and indeed new areas in their local environment.

For Stacey there was a passion for discovering different areas, rather than collecting the Beat Boxes, “Getting to discover places I didn’t know. We did Cupcake Lane and the abandoned station is a teashop. One day I thought, “Let’s try going a little further.” I hadn’t realised there was a massive park at the back of Newbridge. I walked all around there and found the Wolves training ground.” Although for Jennine and Rob there was a sense of achievement in ‘collecting’ the boxes over the course of the game period, they explained, “My little boy was looking at the map, marking it off and circling where he’d been.” (Jennie) and “Being involved in a successful day, it becomes habit. You come home thinking you’ve had a good day. So that’s an addictive feeling. Once we knew we could map up which ones we were going to, then we could do that one on the way to other places.” (Rob).

### Accessibility

The simplistic and inclusive nature of the intervention seemed to appeal broadly. Participants felt they could play in any way they wanted, whether completing a circuit close to their house or travelling further afield to tap boxes in the wider vicinity of the game area. They felt they didn’t need specialist equipment or clothing and that participant didn’t carry a financial burden.

Ed and Rob, respectively, explained how the game could be incorporated into daily routine without any cost, mentioning “You’ve got family and work commitments. You can play the game on your doorstep” and “There's nothing more accessible than walking around. Clubs usually have a membership or some kind of cost involved. This is accessible for anyone.” Supporting these arguments, Maggie revealed how Beat the Street had altered her regular travel behaviour for shopping: “If I go to Asda (local supermarket) in Wolverhampton I walk down there and catch the bus back. With Beat the Street, there was the incentive to carry my shopping and walk back.”

### Awareness

The game aimed to promote health by stealth where the health benefits of physical activity weren’t promoted. However, taking part in the program made individuals self-aware of the benefits the program, which facilitated walking, cycling and communication between residents, was having on their physical and emotional wellbeing. This process of self-discovery motivated participants to take other steps to maintain or further these benefits, such as joining a traditional gym or spending more time outdoors.

Lucy and Katherine both talked to the group about the influence it had had on their physical activity: “Yes, it encouraged me to do more of that [cycling]. I can see the benefit of what I’ve done, and I don’t want to stop doing it. I want to do it more.” (Lucy) and “I went back to the gym. It prompted me to think about how important it was. We live too far from the school to walk the whole way. With Beat the Street we started walking partway. I felt healthier, more toned and it reminded me how I used to feel. I never used to care about people looking at me and I’d started to care so I wanted to get back to how I used to feel.” (Katherine). For Sarah, however, the behaviour change came from an awareness of nature, rather than physical activity solely “I’m still walking a lot. I spend more time outdoors because of Beat the Street. I think there’s more awareness of the benefits of being out in the fresh air.”

### Barriers to engagement

There were several varied motivations for engaging with the intervention, however, there were also number of potential barriers to both engagement during the game period and sustained participation in physical activity, post-game. These potential barriers were mostly reported by participants who engaged with Beat the Street through their child(ren), who often cited practical barriers such as safety or the game set-up, i.e. proximity of beat boxes to each other. For instance, Noel: “This year [it was the second year of the game in this area] they were outside the park and across the road. So that’s dangerous to take them [the children] across the road” and Alan: “To be more kid friendly, there would need to be more [beat boxes] that were closer together. If they’ve got to walk half a mile to get two, they won’t”. Those, who were engaged directly in the intervention also provided recommendations for improving the programme, which might help with engagement. For instance, Chris: “The boxes were the same colour as the lamppost, and you'd go past it. Put fluorescent tape on it.” and Jennie: “Sometimes the little ones would fight to tap my card. Sometimes they didn’t all work. Some of the boxes didn’t work.”

### Theoretical contributions

The current study indicated several varied motivations for participation in the intervention which may have been activated by distinct game mechanisms. Key motivating factors for engagement with the intervention were:

1. Collective reward, acquiring points for collective gain (i.e. a charity or school).
2. Social influence, taking part in an activity with shared common goals with family, friends and/or colleagues.
3. Exploration, discovering the local area, finding new routes and greenspaces, and orienteering to ‘collect’ ‘Beat Boxes’.
4. Game reinvention, for instance, using the game as motivation for activity as part of a patient’s treatment plan or using it as motivation for lunchtime activity in the workplace.
5. Accessibility, awareness of the ease by which participation could be incorporated into daily life and regular routine.
6. Awareness, where participant’s involvement triggered engagement in other sustainable physical activity, such as going back to the gym, and changing dietary behaviours which stemmed from an increased awareness of their importance, from engagement in the game.

Findings also revealed several barriers to participation which need to be addressed to maximise future engagement. These barriers were mostly expressed by parents who had concerns about child safety, a finding consistent with other outdoor gamification-based interventions. [26] However, there were also broader practical barriers specific to the current intervention design such as placement and visibility of the Beat Box scanners. Both of these are important considerations for future interventions of this nature.

There are several game mechanisms which may have engaged participants via the varied motivations presented above. Firstly, the game used a points-based system centred on collective reward. Previous research has shown collective rather than individual extrinsic rewards are more powerful motivations for changing behaviour. [27,28] Second, the game facilitates social action by grouping players into teams, which may have functioned to improve overall game experience and to reduce perceived social isolation. [15] Finally, the game relies on individuals exploring their local environment, seeking out boxes, map reading and ‘completing’ the number of boxes visited. This aspect of the intervention activates an alternative motive to point accumulation and potentially allows the intervention to engage a distinct type of player. Whilst gamification-based initiatives have the potential to engage individuals through a plethora of varied motivations, they also present several barriers concerning the set-up of the game. As such, providers should consult and indeed implement co-production approaches with prospective players prior to game implementation to maximise potential for community engagement.

### Practical implications

There are several important practical implications from this qualitative research. Whilst the current findings cannot be generalised to the 25,790 participants that took part in the intervention, combined with other recent qualitative inquiries of gamification-based programmes, they suggest that the mass appeal of these initiatives could stem from the many, and varied, motivations which they are able to cater for. Firstly, this research implies that interventions, both individualistic and community-wide, need to be simple to engage with, social in nature and facilitate social interaction, co-operation, encourage outdoor activity, and provide a degree of healthy competition, where possible. This argument is supported by a recent systematic review of physical activity messaging which suggests messages should be framed positively and highlight short-term outcomes, specifically relating to social and mental health. [29]

Second, programs should be as inclusive as possible, where individuals of different abilities can compete, or participate with no or minimal cost and without the requirement of specialist clothing or equipment. Again, using Parkrun as an example, its the growing success highlights how making activities easy, financially accessible and inclusive to individuals of all backgrounds may be crucial to generating mass participation in regular physical activity. Third, intervention providers could benefit from recognising the allure of approaches which tap into the so called biophilic instinct to be outdoors. Other approaches, such as incentives to collect or accumulate achievements(i.e. treasure hunts and charitable challenge type events, such as mud runners, triathlons and fun runs) could also be capitalised on for initial engagement techniques to such activity. This research raises the question whether there is a need to reframe the messaging used to publicise and promote the benefits of physical activity or risks of inactivity. This research suggests that allowing individuals the time, autonomy and opportunity to discover the benefits for themselves may produce more salient results than traditional health messaging has achieved. Finally, this research suggests there is a need to engage the public, through proper and legitimate co-production, in the design of interventions, where possible, to limit the avoidable barriers to participation identified above.

### Future directions

The findings from this exploratory qualitative study, combined with other similar investigations of gamification approaches, demonstrate how gamification can tap into several distinct motivations likely to be responsible for the engagement of substantial portions of the population. [14,19] What this study was unable to establish, and indeed has not yet been investigated in the gamification literature to date, is how such approaches can lead to longer term, sustained behaviour change, at a population level. To date, only short-term pilot evaluations and longer-term evaluations with self-selecting participants without a control group have been undertaken. Controlled trials remain unexplored partly due to the newness of such approaches in this area of public health. [15,16,30,31,32,33] There is need to understand the potential impact of this type of intervention in cultures outside of the United Kingdom. It is plausible that weather and existing patterns of physical activity and active travel may impact upon intervention uptake and effectiveness. Furthermore, little is known about the factors pertinent to successful implementation and a process evaluation of these interventions would be particularly worthwhile. Finally, these motivations may be unique to adults and qualitative research into the motives and barriers of children is necessary given that they are a key target demographic of interventions of this kind.

### Conclusion

These findings provide insight not previously captured in research to date, into the experiences of participants following engagement with a gamification-based community-wide physical activity intervention. Gamification-based approaches can engage individuals through several varied motivations which is likely to be a key underlying factor enabling these interventions to engage substantial numbers of people. However, there is a pertinent need for large-scale controlled trials to reveal the extent of behaviour change achieved through gamification based public health interventions such as Beat the Street. There is also a need for such interventions to be part of a wider whole systems approach to addressing physical inactivity
